# Signaling pathways required for macrophage scavenger receptor-mediated phagocytosis: analysis by scanning cytometry

**DOI:** 10.1186/1465-9921-9-59

**Published:** 2008-08-07

**Authors:** Timothy H Sulahian, Amy Imrich, Glen DeLoid, Aaron R Winkler, Lester Kobzik

**Affiliations:** 1 Harvard School of Public Health, Molecular and Integrative Physiological Sciences Program, 655 Huntington Ave, Building II, 2nd Floor, Boston, MA 02115, USA; 2 Department of Inflammation, Wyeth Research, 35 Cambridge Park Dr., Cambridge, MA 02140, USA; 3 Department of Pharmacology and Experimental Therapeutics, Boston University School of Medicine, 715 Albany St., L-603, Boston, MA 02118, USA

## Abstract

**Background:**

Scavenger receptors are important components of the innate immune system in the lung, allowing alveolar macrophages to bind and phagocytose numerous unopsonized targets. Mice with genetic deletions of scavenger receptors, such as SR-A and MARCO, are susceptible to infection or inflammation from inhaled pathogens or dusts. However, the signaling pathways required for scavenger receptor-mediated phagocytosis of unopsonized particles have not been characterized.

**Methods:**

We developed a scanning cytometry-based high-throughput assay of macrophage phagocytosis that quantitates bound and internalized unopsonized latex beads. This assay allowed the testing of a panel of signaling inhibitors which have previously been shown to target opsonin-dependent phagocytosis for their effect on unopsonized bead uptake by human *in vitro*-derived alveolar macrophage-like cells. The non-selective scavenger receptor inhibitor poly(I) and the actin destabilizer cytochalasin D were used to validate the assay and caused near complete abrogation of bead binding and internalization, respectively.

**Results:**

Microtubule destabilization using nocodazole dramatically inhibited bead internalization. Internalization was also significantly reduced by inhibitors of tyrosine kinases (genistein and herbimycin A), protein kinase C (staurosporine, chelerythrine chloride and Gö 6976), phosphoinositide-3 kinase (LY294002 and wortmannin), and the JNK and ERK pathways. In contrast, inhibition of phospholipase C by U-73122 had no effect.

**Conclusion:**

These data indicate the utility of scanning cytometry for the analysis of phagocytosis and that phagocytosis of unopsonized particles has both shared and distinct features when compared to opsonin-mediated phagocytosis.

## Background

Lung infection is responsible for more disability-adjusted life years lost than any other disease [1] and high levels of inhaled dusts have been linked in several epidemiological studies to increases in ear and airway infections, cardiovascular disease, lung cancer and mortality [2-5]. Alveolar macrophages (AMs) are a first line of defense against inhaled bacteria and environmental dusts. Therefore, understanding the mechanism by which AMs defend against inhaled insults is crucial. Since contact with inhaled particles often takes place before an antibody response has occurred or with particles for which specific antibodies are not readily made, the AM relies on innate receptors to recognize inhaled particles.

Scavenger receptors (SRs) are a key component of the innate immune system. In addition to their well-known role in low-density lipoprotein metabolism, SRs play a critical role in AM clearance of inhaled particles by binding and allowing the cells to internalize unopsonized microorganisms, apoptotic bodies and environmental dusts [6,7]. General blockade of SRs using polyanionic inhibitors results in a dramatic reduction of AM uptake of residual oil fly ash, ambient air particles, diesel dust, iron oxide, titanium dioxide, silica, *Escherichia coli *and *Staphylococcus aureus *[8-11]. Specific blockade and transfection of members of the SR family have shown these receptors to be capable of binding several Gram-positive and Gram-negative bacteria as well as isolated lipopolysaccharide and lipotechoic acid [12-21]. In addition, mice deficient in SR-A or MARCO demonstrate reduced bacterial clearance, increased pulmonary inflammation and increased mortality following an intranasal challenge with *Streptococcus Pneumoniae *[10,22]. Furthermore, MARCO can bind CpG DNA [23], whereas blockade of MARCO with a monoclonal antibody dramatically reduces AM uptake of titanium dioxide, iron oxide, silica and latex beads [24,22,25]. SR-A and MARCO, therefore, are clearly critical components of pulmonary host defense. However, it is important to point out that AMs also express several other less well-characterized SRs including LOX-1, SR-PSOX and SRCL [10]. These SRs are capable of binding bacteria [26-28] and might also contribute to the AM response to inhaled insults.

While it is clear that SR-initiated uptake of inhaled particles is critically important for lung defense, it is currently not known which signaling pathways are necessary for SR-mediated phagocytosis. In contrast, phagocytosis of opsonized particles (via Fc or complement receptors) has been well characterized [29]. Many characteristics of opsonin-mediated phagocytosis are shared by both Fc and complement receptors (such as signaling by tyrosine kinase, protein kinase C (PKC), phosphoinositide-3 kinase (PI-3K), mitogen activated protein kinases (MAPK) and phospholipase Cγ (PLCγ)). In contrast, some characteristics are unique to one receptor pathway (such as sensitivity of complement-mediated uptake to microtubule inhibitors) [30]. Many of these opsonin-mediated phagocytic signaling pathways have also been implicated in non-phagocytic SR-mediated responses such as cytokine production and lipoprotein endocytosis [31-38]. We hypothesized that these pathways would also be necessary for SR-mediated phagocytosis. To test this, we employed a battery of well-established signaling inhibitors and a novel high-throughput fluorescence phagocytosis assay.

AMs are known to express a wide array of SRs with overlapping ligand specificities. Therefore, it is likely that inhaled particles are simultaneously bound by multiple SR family members. Since the underlying biology of the particle-AM interaction is more complicated than a simple one ligand/one receptor interaction, we chose a target particle (latex spheres) that likewise binds multiple SRs to more closely model the true physiology of particle-AM interactions. It should be noted that the latex sphere has long been used as a model for inhaled particulates and is similar to 'real world' particles in terms of its SR-mediated uptake by AM [10,39,9,25,42].

## Methods

### Cell isolation, differentiation and characterization

Discarded platelet apheresis collars were obtained from the Kraft Family Blood Donor Center at the Dana-Farber Cancer Institute (Boston, MA, USA). Buffy coats were harvested from these collars and enriched for monocytes using the RosetteSep Monocyte Enrichment kit (Stem Cell Technologies, Vancouver, BC, Canada). Monocytes were then cultured in Vuelife bags (American Fluoroseal, Gaithersburg, MD, USA) for 11 days at 5% CO_2 _and 37°C in RPMI/10% FBS/20 μg/ml gentamicin supplemented with 20 ng/ml human granulocyte/macrophage-colony stimulating factor (GM-CSF, Peprotech, Rocky Hill, NJ, USA). GM-CSF matured MØ (GM-MØ) were then harvested and resuspended at 1 × 10^6^/ml in RPMI/10% FBS. 1 × 10^5 ^cells were dispensed into black-walled 96 well Micro-Clear plates (Greiner Bio-One, Monroe, NC, USA). For some experiments, the number of cells per well was altered but the volume remained constant. After plating and adherence, GM-MØ were incubated for 40–44 hours, and then used to measure particle binding and internalization.

Some GM-MØ were characterized by flow cytometry before being plated for experiments. Cells were stained with anti-PSOX (10 μg/ml, provided by Dr. Kimihisa Ichikawa, Sankyo, Tokyo, Japan), anti-LOX-1 (10 μg/ml, Cell Sciences, Inc., Canton, MA, USA), anti-SR-A (10 μg/ml, provided by Dr. Motohiro Takeya, Kumamoto University, Kumamoto, Japan), anti-CD68 (10 μg/ml, Dako, Carpinteria, CA, USA), anti-CD14 (3.3 μg/ml), anti-HLA-DR (10 μg/ml), anti-HLA-DQ (10 μg/ml) or equal concentrations of isotype matched control antibodies (all from BD Biosciences, Rockville, MD, USA) in PBS with 2 mg/ml bovine serum albumin (BSA) and 4 mg/ml human IgG (both from Sigma, St. Louis, MO, USA). This step was followed by staining with 20 μg/ml Alexafluor 488 labeled F(ab')_2 _goat anti-mouse antibodies (Invitrogen, Carlsbad, CA, USA) and fixation in PBS with 1% paraformaldehyde. Other cells were stained with 10 μg/ml PLK-1 (anti-MARCO [10]) or control IgG that had been biotinylated using biotin-X-NHS (Calbiochem, San Diego, CA, USA). This was followed by secondary staining with 7.5 μg/ml streptavidin-phycoerythrin (Invitrogen) and fixation as described above. Cellular fluorescence was measured using a Coulter Epics Elite flow cytometer (Beckman Coulter, Miami, FL, USA).

Cells were also evaluated for their ability to bind unopsonized latex beads in the presence or absence of SR inhibitors. One hundred microliters of GM-MØ (suspended at 2 × 10^6^/ml in HBSS/0.3% BSA) were plated in each well of a low adherence 96-well plate (Corning, Corning NY, USA). One hundred microliters of 20 μg/ml polyinosinic acid (poly (I)), 20 μg/ml chondroitin sulfate (both from Sigma), 20 μg/ml PLK-1 mAb or 20 μg/ml mIgG_3 _isotype control (eBioscience, San Diego, CA, USA) were added and cells were allowed to incubate for 10 minutes at 37°C. One hundred microliters of green fluorescent latex beads (1 μm, Invitrogen) were added at a concentration of 1 × 10^8^/ml in HBSS/0.3%BSA with or without 10 μg/ml poly(I), 10 μg/ml chondroitin sulfate, 10 μg/ml PLK-1 or 10 μg/ml mIgG_3_. This corresponds to a 50:1 bead to cell ratio. Cells were incubated for 30 minutes at 37°C, with gentle pipetting every 10 minutes to resuspend the cells and beads. After incubation, the assay was stopped by chilling cells on ice and analyzing fluorescence by flow cytometry.

For mouse studies, primary AMs were isolated from C57BL/6J mice (The Jackson Laboratory, Bar Harbor, ME, USA). Immediately before bronchialveolar lavage, mice were euthanized by an overdose of Phenobarbital. The lungs were lavaged six times with 0.8 ml of ice-cold PBS. Cell purity and yield was determined using a hemocytometer. Murine AMs were cultured in black-walled 96 well Micro-Clear plates in RPMI/10% FBS for 40–44 hours before phagocytosis assays were performed as described for GM-MØ.

### Preparation of biotinylated latex beads

Biotin-BSA was generated by incubating 50 mg of tissue culture grade BSA (Sigma) with 30 mg biotin-X-NHS in 10 ml PBS for one hour at room temperature. Unconjugated biotin was removed by extensive dialysis. Green fluorescent carboxylated latex beads (1 μm, Invitrogen) were centrifuged at high speed and washed twice in 2-(N-morpholino)ethanesulfonic acid (MES, Calbiochem) buffer (19.2 mg/ml, pH 6.0). Beads were suspended at 5 × 10^9 ^per ml in MES buffer. Water-soluble carbodimide (WSC, Calbiochem) was freshly dissolved in MES buffer and beads were incubated at room temperature for one hour with 10 mg/ml WSC. Beads were washed twice in 0.5 × PBS and resuspended in water. An equal volume of biotin-BSA was added for a final concentration of 2 mg/ml BSA in 0.5 × PBS. Beads were incubated overnight at room temperature and then centrifuged at high speed. Beads were then resuspended in 0.5 × PBS with 40 mM glycine and incubated for one hour. Finally, beads were washed twice in PBS containing 0.2% BSA and 0.01% sodium azide and stored at 4°C.

### Internalization assay

All reagents and buffers were at room temperature when added to cells and all incubations were performed in warm (37°C) humid air unless otherwise noted. All fluorescent dyes were purchased from Invitrogen. Cells were incubated with CellTracker Blue at 100 μM in HBSS with Ca^++ ^and Mg^++ ^(Cambrex, East Rutherford, NJ, USA) for 40 minutes followed by a 30 minute recovery period in assay buffer (HBSS/0.3% BSA). Inhibitors (Table 1) or DMSO were then added for 20 minutes. Poly(I), cytochalasin D, nocodazole, staurosporine, wortmannin and herbimycin A were purchased from Sigma. All other inhibitors were purchased from Calbiochem. GM-MØ were then incubated for 20 minutes with bead suspension (2 × 10^8 ^beads/ml) +/- inhibitors for bead binding and internalization. Cells were then washed 2 × 250 μl with assay buffer, covered with fresh buffer +/- inhibitors and incubated for an additional 20 minutes to allow for further bead internalization (the cells were, therefore, incubated with inhibitors for a grand total of 60 minutes). After this the cells were washed and extracellular beads were labeled on ice for 30 minutes using streptavidin-Texas Red (20 μg/ml in assay buffer). After a final wash with 250 μl assay buffer, cells were fixed with 4% paraformaldehyde in PBS. The fixative was removed after 30 minutes and cell nuclei were stained for 30 minutes with 3 μg/ml of Hoechst 33342. The Hoechst dye was then removed and wells were filled with 100 μl of 4% paraformaldehyde in PBS for storage.

**Table 1:** Summary of Inhibitors Tested.

Inhibitor	Target	Final Concentration	Vehicle
Poly(I)	SR binding	10 μg/ml	PBS
Cytochalasin D	filamentous actin	15 μM	DMSO
Nocodazole	microtubules	25 μM	DMSO
Staurosporine	protein kinases	1 μM	DMSO
Chelerythrine Cl	PKC	25 μM	DMSO
Gö 6976	PKC	10 μM	DMSO
Wortmannin	PI-3K	0.04 μM	DMSO
LY294002	PI-3K	200 μM	DMSO
Genistein	tyrosine kinases	100 μM	DMSO
Herbimycin A	tyrosine kinases	80 μM	DMSO
MEK inhibitor I	MEK	200 μM	DMSO
JNK inhibitor I	JNK	4 μM	PBS
JNK control	inactive analog of JNK inhibitor I	4 μM	PBS
U-73122	phospholipase C	10 μM	DMSO
U-73343	inactive analog of U-73122	10 μM	DMSO

### Image Acquisition and Data Analysis

Images of adherent cells were collected using the Pathway HT bioimager (BD Biosciences). Cells were both illuminated through and fluorescence emission was collected from the bottom of the plate using a 20 × NA075 lens (Olympus, Center Valley, PA, USA) and a field size of approximately 300 μm square. All images were collected using flat field correction and 2 × 2 binning of pixels. Auto focus was carried out using the fluorescence emission of Hoechst and CellTracker Blue, which share the same excitation and emission spectra. Confocal images of bead fluorescence (488 BP excitation, 515 LP dichroic, 515 LP emission filters), Texas Red (560 BP excitation, 595 LP dichroic, 645 LP emission filters) and Hoechst/CellTracker Blue (380 BP excitation, 400 LP dichroic, 435 LP emission filters) were collected every 1.7 μm for a total of 10 sections. The dyes were illuminated sequentially and the confocal images collected were collapsed, creating new images with clear definition of all beads within each cell.

Cell segmentation for each image was achieved using a combination of the Hoechst signal (to identify single cells) and the CellTracker Blue signal (to define the cell borders). Using the collapsed stacks of confocal images, software was developed to define the cells (blue emission image), count the number of beads per cell (green emission image) and determine if the beads are outside the cell (red emission image) using custom software developed in MATLAB (The Mathworks, Inc., Natick, MA, USA). Hoechst/CellTracker Blue images were processed to reduce noise, enhance contrast and correct for non-uniform field brightness. A gradient-facilitated watershed segmentation algorithm was used to identify and label individual cells. Cell sizes (profile areas) were calculated as the number of pixels in segmented cell objects (collapsed stack images). Cell volumes were calculated as the sum of the cell profile areas of the individual confocal images comprising collapsed stacks. Green fluorescent (all beads) and red fluorescent (external beads) images were sharpened and contrast enhanced. Watershed segmentation was used to identify and label individual bead objects. Labeled bead objects within the "all beads" image were classified as "internal" if they had less than 20% overlap with an external bead object. Bead objects sharing one or more pixel with any cell object were considered to be associated with that cell. All partial cell images along the edges of the field were omitted from analysis.

Bead binding was calculated as the average number of cell-associated beads per cell. Typically between 1200 and 1800 total cells were counted per donor per condition. Percent internalization was calculated as the number of internalized beads divided by the total number of cell-associated beads for each cell, then multiplied by 100. Significant differences were calculated for the poly(I) data using Students paired *t*-test. For the cell density data, the Spearman correlation test was performed. For all other data, significant differences were calculated using one-way ANOVA followed by Bonferroni's multiple comparison of all means. An unpaired ANOVA was used in the analysis of the protein tyrosine kinase data in Figure 8. For all other data, a paired ANOVA was used. Prism 4 for the Macintosh (Graphpad Software, San Diego, CA, USA) was used for all graphing and statistical calculations.

## Results

### Characterization of GM-MØ

Monocytes are typically matured into MØ *in vitro *using M-CSF. However, AM are unusual in that they require GM-CSF, but not M-CSF, for their development *in vivo *[43-47]. Therefore, we followed the GM-CSF-based differentiation protocol of Akagawa, *et al*., designed to produce monocyte-derived MØ with a distinctly AM-like phenotype (GM-MØ) [48]. Both AM and GM-MØ have been shown to produce lower levels of H_2_O_2_, express higher levels of catalase and are more resistant to H_2_O_2 _toxicity when compared to M-CSF derived MØ. Furthermore, AM and GM-MØ (but not M-CSF derived MØ) express HLA-DQ and are resistant to HIV infection, but susceptible to *Mycobacterium tuberculosis *infection [48,49]. Finally, we are confident that GM-MØ are an appropriate model for primary AMs in that several of the inhibitors described in this communication (genistein, herbimycin A, wortmannin, nocodazole and staurosporine) were also tested for their ability to inhibit phagocytosis of beads by primary murine AM. In all cases, the results were comparable to those obtained using GM-MØ (data not shown).

**Figure 1:**
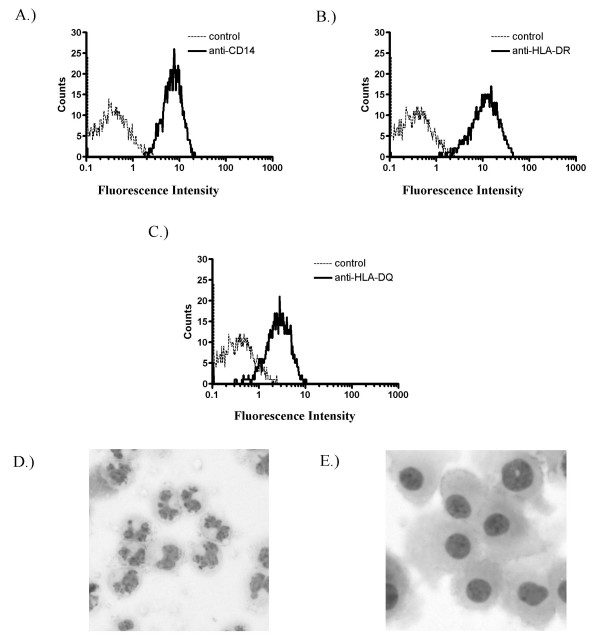
**Characterization of GM-MØ**. CD14 (A), HLA-DR (B) and HLA-DQ (C) expression were evaluated by flow cytometry. Solid lines represent the fluorescence of stained cells, while dashed lines represent the results from control antibodies. Data are representative of experiments performed on cells from three donors. Cytocentrifuge preparations illustrating monocyte and macrophage morphology before (D) and after (E) maturation with GM-CSF were captured at equal magnification (200×).

**Figure 2:**
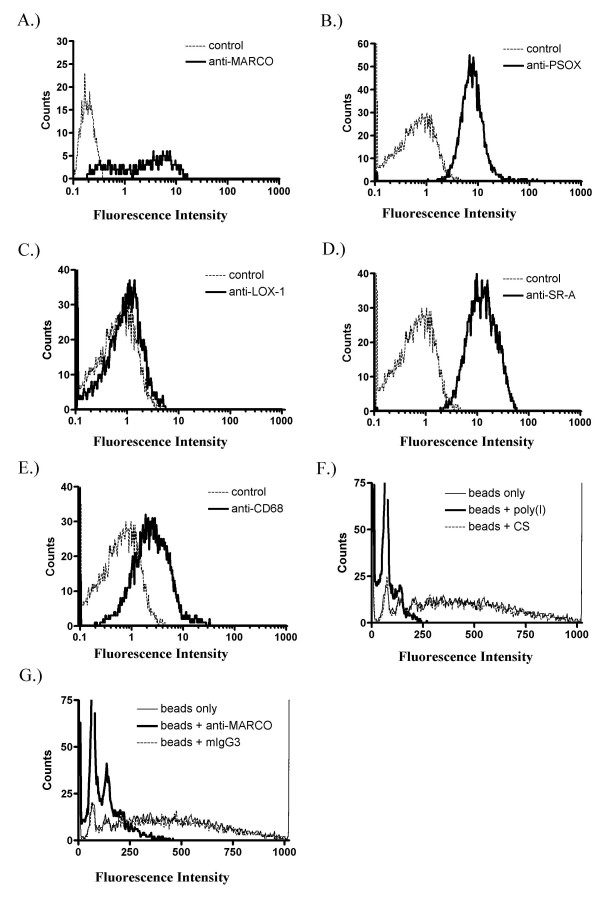
**Characterization of SRs expressed by GM-MØ**. MARCO (A), PSOX (B), LOX-1 (C), SR-A (D) and CD68 (E) expression as well as fluorescent bead binding in the presence of poly(I) (F) or an anti-MARCO mAb (G) were evaluated by flow cytometry. Data are representative of experiments performed on cells from three (A-E) or five (F and G) donors.

It should be noted that, unlike murine bone marrow, incubation of human monocytes with GM-CSF alone does not produce dendritic cells, as evidenced by the morphology and surface marker expression of GM-MØ. GM-MØ were harvested after 11 days of culture in GM-CSF-supplemented media and immunolabeled to measure surface expression of general macrophage markers as well as markers which can differentiate between alveolar/GM-MØ and the more traditional M-CSF matured MØ. As shown in Figure 1, greater than 90% of GM-MØ stain positive for the MØ surface proteins CD14 and HLA-DR and demonstrate a MØ-like morphology when analyzed by light microscopy, confirming their identity as MØ. These cells are also positive for both HLA-DQ and MARCO (Figure 2), a phenotype consistent with both GM-MØ and primary AMs [10,48,25,52]. In addition, GM-MØ were labeled for SRs known to be present on primary AMs [53,54,10]. As shown in Figure 2, GM-MØ are weakly positive for CD68 and strongly positive for MARCO, PSOX and SR-A.

Our findings also confirm that SRs are involved in the binding of unopsonized latex beads. As shown in Figures 2F and 2G, bead uptake is dramatically inhibited by either the broad SR blocker poly(I) or the MARCO-specific SR blocker mAb PLK-1. These agents reduced the fluorescent bead signal by 80% and 62% respectively, whereas their control reagents (chondroitin sulfate and mIgG3) had no effect. Taken together, these data suggest that GM-MØ accurately model primary AMs in their expression of a wide range of SRs and that their interaction with unopsonized beads involves MARCO (and likely other SRs as well).

### High throughput direct measurement of phagocytosis

**Figure 3:**
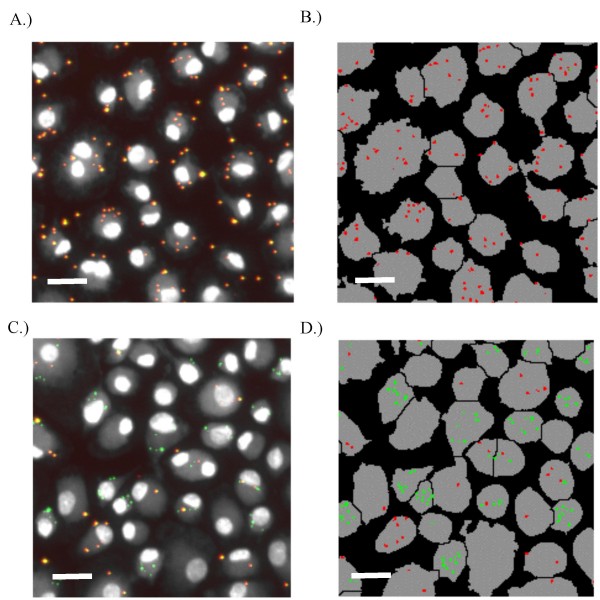
**Binding and phagocytosis assay**. Adherent GM-MØ were treated with cytochalasin D (A and B) to block internalization or vehicle control (DMSO, C and D) before incubation with biotinylated green fluorescent latex beads and labeling of extracellular beads with streptavidin-Texas Red. Images obtained by scanning cytometry show intracellular beads in green, while extracellular beads are in yellow (due to the colocalization of red and green fluorescence). Panels A and C depict the fluorescence images obtained by the scanning cytometer, while panels B and D depict the same images after automated segmenting and bead identification. Scale bars represent 20 μm.

A high throughput phagocytosis assay was developed to provide rapid and direct measurement of both particle binding and internalization. For this assay, GM-MØ are first incubated with CellTracker Blue, which provides a uniform label of the whole cell to facilitate cytometric identification. The GM-MØ are then allowed to bind and ingest biotinylated green fluorescent latex beads, followed by incubation with streptavidin-Texas Red to label external beads. Analysis with a scanning cytometer produces images in which beads that are bound, but not internalized, are clearly distinguishable from those which are internalized. Figures 3A–D are typical examples of images produced by this technique. In Figures 3A and 3B, phagocytosis has been inhibited by cytochalasin D treatment. As a result, all of the beads are extracellular and appear as yellow, due to the colocalization of red and green fluorescence. In contrast, the cells in Figures 3C and 3D have been allowed to internalize beads. In these images, some beads are extracellular (appearing as yellow) while others have been internalized (appearing as green). The cells in these images can be automatically identified ('segmented') and the number of beads per cell counted using a combination of commercial and custom software (Figure 3B and 3D).

**Figure 4:**
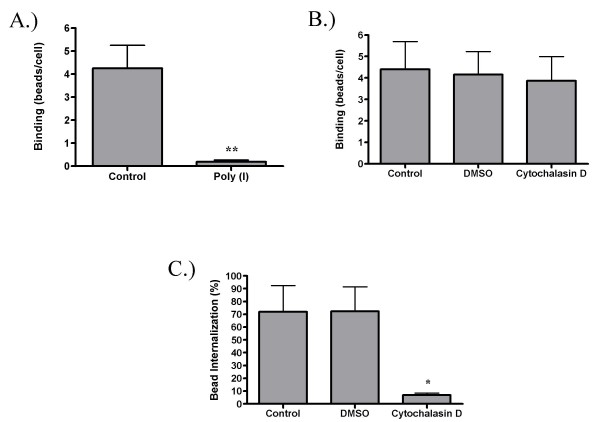
**Quantification of bead binding and internalization**. Adherent GM-MØ were pretreated with either poly(I) (A) or cytochalasin D (B and C) before incubation with fluorescent latex beads. Cells were analyzed for total bead binding (A and B) and percent internalization (C). Bars represent the means of four (A) or three (B and C) donors +/- the standard deviation. For each donor, three fields from each of three replicate wells were analyzed. *p < 0.01 **p < 0.001 when compared to either the control or DMSO conditions.

In order to validate this technique, GM-MØ were cultured with known inhibitors of SR binding and phagocytosis before being incubated with fluorescent beads. We observed a nearly complete (96%) reduction in the number of beads bound by cells in the presence of the SR blocker poly(I) (Figure 4A). In contrast, the actin destabilizer cytochalasin D has no effect on total bead binding, but decreases the number of beads internalized by 90% when compared to the DMSO control (Figures 4B and 4C). To compare the results of software image analysis to human quantitation of the same images, beads per cell were manually counted for 50 cells in both the control and cytochalasin D treated conditions. These results were quite similar to those obtained by software analysis and are shown in Table 2. Hence, our software quantification technique is capable of accurately counting and distinguishing between beads that have been internalized and beads that have been bound, but not internalized.

**Table 2:** Manual vs. Computer Enumeration of Cell-Associated Beads.

	Internalized Beads		Extracellular Beads	
	Control	Cytochalasin D	Control	Cytochalasin D

Manual	126	10	57	221
Computer	130	11	69	234

### Binding and internalization are differentially affected by cell density

**Figure 5:**
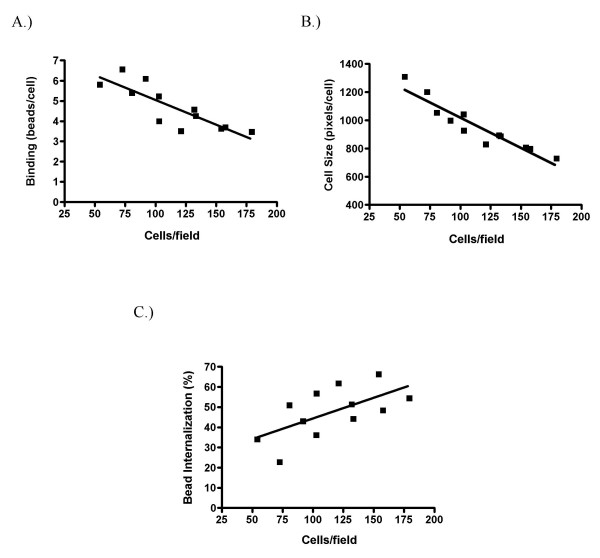
**Bead binding and internalization are cell density dependent**. GM-MØ were plated at 5 × 10^4 ^to 1.25 × 10^5 ^cells/well and cultured overnight before incubation with fluorescent latex beads. At the end of the experiment, cell density for each well was calculated as the average number of cells per field from three fields. Cells were analyzed for total bead binding (A), cell size (B) and bead internalization (C). Points represent the means of three wells each. Lines represent the best-fit linear regression. Data are pooled from three donors.

To determine the optimal cell concentration for this assay, we compared results using a range of cell densities. The data collected indicate that cell density affects cell size, and has considerable and opposing effects on bead binding and internalization (Figure 5). Higher plating densities are associated with reduced cell size, as measured by pixels per cell profile in collapsed stack images, (r = -0.972, p < 0.001). Comparison of cell sizes and cell volumes calculated from confocal slices confirmed that cell size accurately reflects cell volume (n = 95 cells, r = 0.971, p < 0.0001, data not shown). As cell density increases, the number of beads bound per cell decreases significantly (r = -0.853, p < 0.001). This could be due to reduced cell size and/or increased cell-to-cell contact, thereby reducing the cellular surface area available for bead binding. In contrast, increasing the cell density dramatically augments the percentage of bound beads that are internalized (e.g., increasing the density of the cells from 54 to 180 cells per field resulted in a 60% increase in the percentage of internalized beads (r = 0.622, p < 0.05)), an observation that cannot easily be explained by a reduction in cell size. Taken together, these data indicate that the density used for in vitro analysis of GM-MØ has a significant influence on phagocytic parameters. For all subsequent experiments, cells were plated at 1 × 10^5 ^cells per well.

### Microtubule destabilization inhibits SR mediated internalization

**Figure 6:**
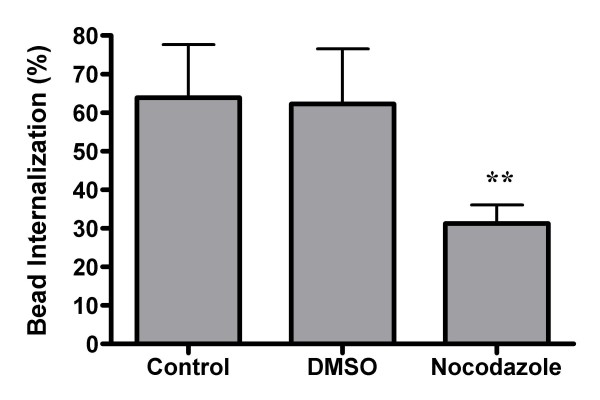
**Microtubule destabilization inhibits SR mediated phagocytosis**. Adherent GM-MØ were pretreated with nocodazole before incubation with fluorescent latex beads. Cells were analyzed for the percent internalization of bound beads. Bars represent the means of five donors +/- the standard deviation. For each donor, three fields from each of three replicate wells were analyzed. **p < 0.001 when compared to either the control or DMSO conditions.

Although filamentous actin is required for phagocytosis in general, the requirement for microtubules depends upon which phagocytic receptor is involved. For example, inhibiting microtubule function blocks complement receptor-mediated, but not Fc receptor-mediated, particle internalization [30,55]. In order to determine if SR-mediated phagocytosis requires microtubules, GM-MØ were analyzed for their ability to bind and internalize latex beads in the presence of the microtubule destabilizer nocodazole. Nocodazole treatment has no effect on the total number of beads bound per cell (data not shown), suggesting that SRs do not require microtubules for particle binding. In contrast, nocodazole treatment reduces the proportion of internalized beads by 50% when compared to the DMSO control (Figure 6). We conclude that SR-mediated internalization is similar to complement receptor-mediated phagocytosis in that they both require functional microtubules.

### Effect of signaling pathway inhibitors on SR-mediated phagocytosis

A large number of signaling molecules have been implicated in MØ phagocytosis [56,57]. However, most of this work has been performed using IgG or (to a lesser extent) complement opsonized particles. Very little is known about which signaling pathways are required for SR-mediated phagocytosis. Our strategy was to analyze these pathways using a panel of relevant pharmacologic inhibitors, an approach facilitated by the high throughput assay described above.

**Figure 7:**
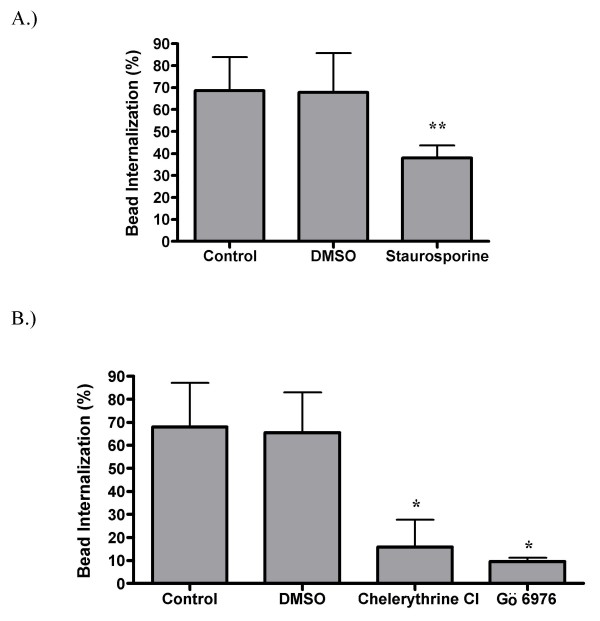
**Protein kinase C blockers inhibit SR mediated phagocytosis**. Adherent GM-MØ were pretreated with either staurosporine (A), chelerythryine chloride or Gö 6976 (B) before incubation with fluorescent latex beads. Cells were analyzed for the percent internalization of bound beads. Bars represent the means of five (A) or three (B) donors +/- the standard deviation. For each donor, three fields from each of three replicate wells were analyzed. *p < 0.01 and **p < 0.001 when compared to either the control or DMSO conditions.

**Figure 8:**
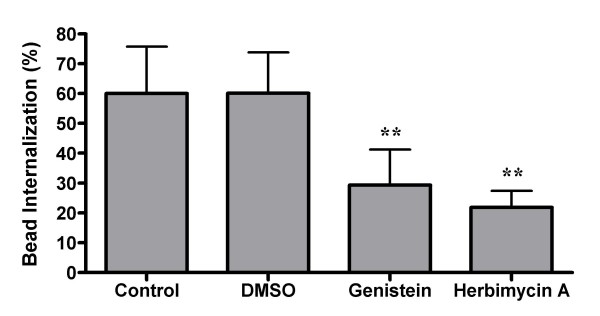
**Protein tyrosine kinase blockers inhibit SR mediated phagocytosis**. Adherent GM-MØ were pretreated with either genistein or herbimycin A before incubation with fluorescent latex beads. Cells were analyzed for the percent internalization of bound beads. Bars represent the means of at least four donors +/- the standard deviation. For each donor, three fields from each of three replicate wells were analyzed. **p < 0.001 when compared to either the control or DMSO conditions.

Tyrosine kinases and PKC are both known to be involved in Fc-receptor mediated phagocytosis [57]. Therefore, we tested the effect of protein tyrosine kinase and PKC inhibitors on SR-mediated phagocytosis (Figures 7 and 8). Inhibition of PKC with staurosporine results in a significant reduction in the number of beads internalized. However, staurosporine is known to inhibit a number of other protein kinases in addition to PKC. In order to definitively show that PKC is required, the PKC specific inhibitors chelerythrine chloride and Gö 6976 were used. These inhibitors cause dramatic (77% and 86%, respectively) reductions in bead internalization. Similarly, treatment with the protein tyrosine kinase inhibitors genistein and herbimycin A result in a 51% and 64% reduction in internalization, respectively. These data show that PKC and tyrosine kinase activities are important for non-opsonic phagocytosis.

**Figure 9:**
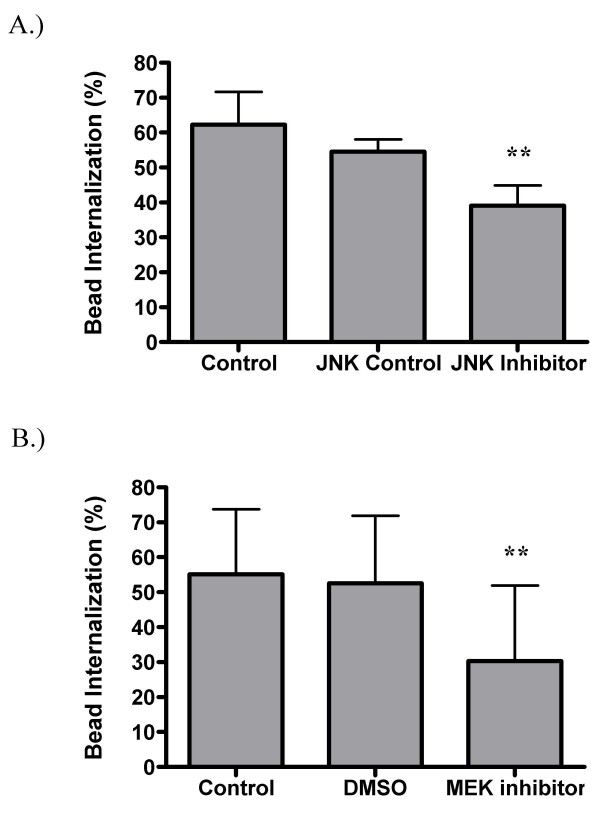
**MAPK blockers inhibit SR mediated phagocytosis**. Adherent GM-MØ were pretreated with inhibitors of either JNK (A) or MEK (B) before incubation with fluorescent latex beads. Cells were analyzed for the percent internalization of bound beads. Bars represent the means of five (A) or six (B) donors +/- the standard deviation. For each donor, three fields from each of three replicate wells were analyzed. **p < 0.001 when compared to either the control, JNK control or DMSO conditions.

The MAPK family of protein kinases is critical for Fc receptor mediated phagocytosis as well as cell cycle progression and a number of other cytoskeletal processes. Since PKC and tyrosine kinases are known to stimulate MAPK [58], inhibitors of the JNK and ERK MAPK pathways were tested for their ability to inhibit SR-mediated phagocytosis. Inhibition of either of these MAPK pathways blocks internalization. The JNK inhibitor reduces bead internalization by 28% while the inactive analog used as a control does not cause a statistically significant reduction (Figure 9A). Inhibition of the ERK pathway was achieved using an inhibitor of the upstream kinase, MEK. Treatment with this inhibitor reduces phagocytosis by 42% when compared to DMSO control (Figure 9B).

**Figure 10:**
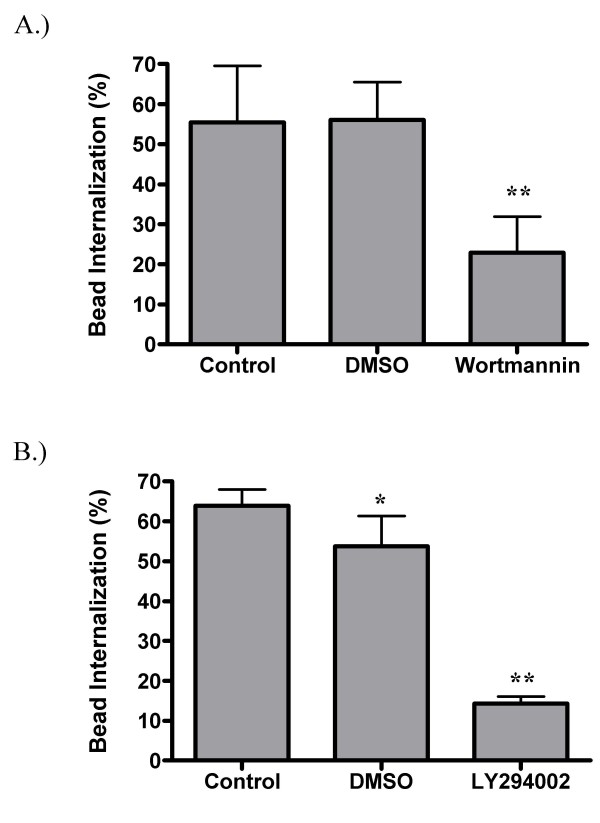
**Effect of PI-3K inhibitors on SR mediated phagocytosis**. Adherent GM-MØ were pretreated with wortmannin (A) or LY294002 (B) before being fed fluorescent latex beads. Cells were analyzed for the percent internalization of bound beads. Bars represent the means of five (A) or three (B) donors +/- the standard deviation. For each donor, three fields from each of three replicate wells were analyzed. **p < 0.001 when compared to either the control or DMSO conditions. *p < 0.05 when compared to the control condition.

**Figure 11:**
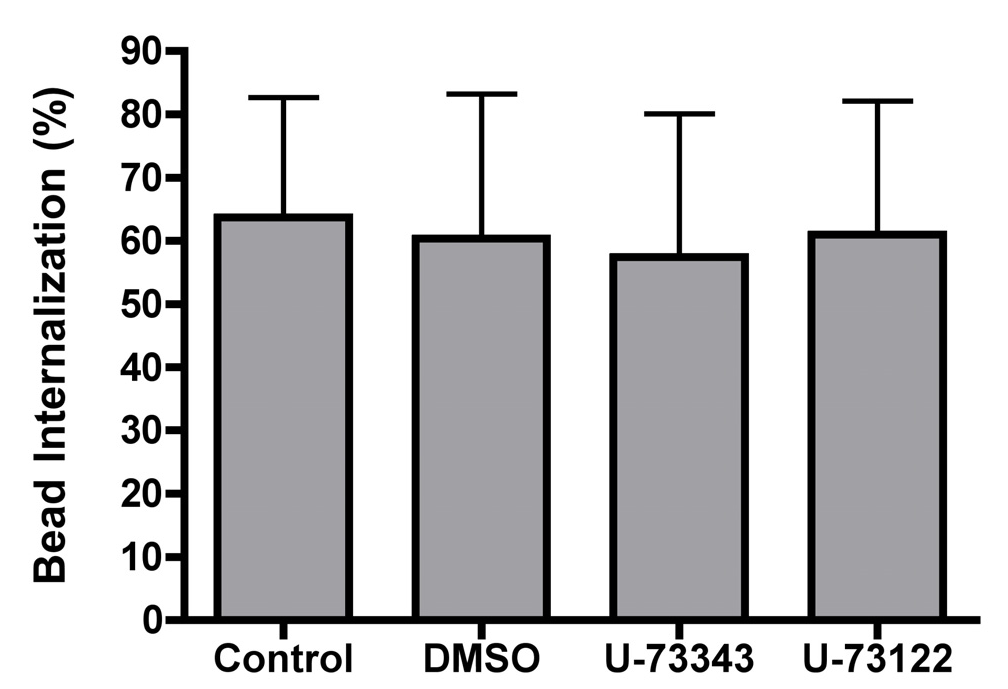
**Effect of a PLCγ inhibitor on SR mediated phagocytosis**. Adherent GM-MØ were pretreated with U-73122 or the inactive analog U-73343 before being fed fluorescent latex beads. Cells were analyzed for the percent internalization of bound beads. Bars represent the means of four donors +/- the standard deviation. For each donor, three fields from each of three replicate wells were analyzed.

In addition to the protein kinases mentioned above, the lipid modifying enzymes PI-3K and PLCγ have also been shown to play a role in MØ phagocytosis [57]. Therefore, the PI-3K inhibitors wortmannin and LY294002 and the PLCγ inhibitor U-73122 were used to block these enzymes before challenging GM-MØ with latex beads As shown in Figure 10A, wortmannin inhibits bead internalization by 59%, while LY294002 causes an even greater inhibition (78%) (Figure 10B). These data demonstrate that PI-3K is required for optimal SR-mediated phagocytosis. However, unlike PI-3K, PLCγ does not appear to be necessary, as U-73122 is unable to block internalization at the concentration tested (Figure 11).

Interestingly, while most of the inhibitors shown in Figures 7, 8, 9, 10, 11 block internalization, none of them have a significant effect on particle binding, cell size or the number of cells per field (data not shown). This indicates that SRs do not require PKC, tyrosine kinase, MAPK, PI-3K or PLCγ signaling to effectively bind unopsonized particles. In addition, the fact that cell size and number are unaffected by the inhibitors used demonstrates that these inhibitors did not affect cell viability. This is confirmed by the observation that the inhibitors used do not alter cellular morphology or increase staining with propidium iodide (data not shown).

## Discussion

While the ligand binding characteristics of SRs have been characterized [6], very little is known about the signaling pathways utilized during SR-mediated phagocytosis. In order to address this, we developed a high-throughput phagocytosis assay capable of distinguishing between internalized and non-internalized cell-associated particles. Using this assay, we tested a battery of signaling inhibitors that are known to block opsonin-mediated phagocytosis for their effect on opsonin-independent phagocytosis. We found that microtubules, PKC, tyrosine kinases, MAPKs and PI-3K are required for optimal SR-mediated phagocytosis. Furthermore, cell density has a significant impact on both particle binding and internalization.

As primary human AM are difficult to obtain in large quantities, we took advantage of a previously published *in vitro *human monocyte differentiation scheme that produces MØ that are phenotypically and physiologically similar to human AM. In order to confirm our findings, we tested a subset of inhibitors (genistein, herbimycin A, wortmannin, nocodazole and staurosporine) for their effect on bead phagocytosis by primary murine AMs. Every inhibitor tested significantly decreased bead internalization. This demonstrates that, at the very least, protein tyrosine kinases, PKC, PI-3K and microtubules are necessary for bead phagocytosis by primary murine AM. These findings are identical to those obtained using GM-MØ and further establish these cells as a useful model of primary AM.

Most currently available phagocytosis assays rely on subtracting the number of particles associated with cells in which internalization has been blocked from the number of particles associated with cells in which internalization has not been blocked. The agents used to block phagocytosis are typically cytoskeletal or mitochondrial poisons such as cytochalasin D or sodium azide (although incubation at low temperature has also been used) [59-61]. Built into these indirect techniques is the assumption that the agent used to block internalization is effective in the particular cells being studied, yet does not alter the number of bound extracellular beads.

In some cases (particularly for receptors of unopsonized targets), this assumption is erroneous, resulting in either an under- or overestimation of particle internalization. For example, our two-color direct approach definitively demonstrates that cytochalasin D is an extremely effective blocker of phagocytosis in GM-MØ (Figure 3D). However, it does not alter the total number of cell-associated beads (Figure 3C). Since the total number of cell-associated beads is the sum of the internalized beads and the beads that have been bound but not internalized, these data indicate that cytochalasin D treatment *does indeed *alter the number of bound extracellular beads under our experimental conditions. In this case, using the indirect single-color technique would have led to a dramatic underestimation of bead internalization by the untreated cells. The opposite problem would have been encountered if a low temperature incubation had been used to block internalization. This is because, unlike opsonized particles, the binding of unopsonized beads is temperature dependent ([42] and our unpublished results).

Given the limitations of the indirect assays mentioned above, we chose to utilize a direct phagocytosis assay based on previously developed two-color fluorescence assays [62,42]. These assays use one intrinsic fluorescent dye to identify all particles and a second non-cell permeable stain applied after internalization to identify particles that have not been internalized. These techniques allow the investigator to distinguish between internalized and extracellular particles without relying on interventions that alter the biology of the cell. While these assays overcome the pitfalls of the indirect assays, they introduce new difficulties for data collection. For example, analysis by flow cytometry can provide exact bead per cell counts for up to three (or perhaps four) cell-associated beads per cell. This is due to the high intensity and low bead-to-bead variability of the intrinsic fluorescent dye. However, at higher bead loads, the absolute number of beads per cell cannot be determined, as the fluorescent peaks begin to overlap [63]. Furthermore, the higher variability and lower intensity of staining with the extracellular dye precludes precise bead per cell counts at even very low bead loads (our unpublished data). As a result of these issues, results are typically reported as a ratio of fluorescence intensities (not absolute bead number) when flow cytometry is used as a read out. The alternative to flow cytometry (counting beads by eye using a fluorescent microscope) is tedious and incompatible with high throughput.

In order to overcome these limitations, we developed a system using scanning cytometer technology that can automatically count the number of beads associated with any given cell and distinguish between internalized and extracellular beads. This system allows the investigator to express his or her data as the number of beads per cell and not simply as fluorescence intensity, even for cells with high bead loads. While this assay is similar in many respects to one recently developed by Steinberg and colleagues for analysis of opsonized phagocytosis [64], it differs in that our method involves collecting a set of confocal images spanning the entire thickness of the cell that are then collapsed into a single image for analysis. This technique allows all of the cell-associated beads to be in focus for the final analysis. In contrast, we have found that using conventional fluorescence microscopy does not allow all of the cell-associated beads to remain in focus simultaneously and therefore excludes some beads from analysis (data not shown).

The confocal-based phagocytosis assay described in this report was used to test the hypothesis that SR-mediated phagocytosis is similar to complement-mediated phagocytosis in respect to its sensitivity to a microtubule inhibitor. Phagocytosis of opsonized particles by Fc or complement receptors share a number of characteristics, including dependence on actin filaments and the accumulation of signaling and actin binding proteins at the site of the forming phagosome [56]. However, fundamental differences exist between these two modes of phagocytosis [65,55,68]. These differences have led some to characterize them as type I (Fc receptor-mediated) and type II (complement receptor-mediated). Microtubule poisons such as nocodazole paralyze complement-mediated, but not Fc receptor-mediated, particle internalization [55,30]. In this report we present the first evidence that SR-mediated phagocytosis exhibits a characteristic of type II phagocytosis in that nocodazole significantly inhibits internalization.

This report is also the first to show that tyrosine kinases, PKC, PI-3K and MAPKs are necessary for SR-mediated phagocytosis by MØ. The requirement for PI-3K and tyrosine kinases is consistent with a recent report showing that PI-3K and the Src kinase Lyn are both required for SR-A-mediated MØ spreading [69]. Furthermore, treatment of MØ cell lines with soluble SR ligands results in the tyrosine phosphorylation of Src kinases, PLCγ and PI-3K as well as a tyrosine kinase dependant activation of PKC [34,33,31,32], suggesting that tyrosine kinase activation may occur relatively early in the SR signaling cascade. Consistent with the inhibition of phagocytosis reported here, inhibition of tyrosine kinases blocks the induction of urokinase-type plasminogen activator (uPA) and IL-1 expression by THP-1 cells in response to SR ligands [31,32]. Similarly, pharmacological blockade of PKC inhibits SR-mediated increases in uPA expression, myelin endocytosis, prostaglandin E2 release and ERK activation [32,36,35].

It is surprising to note that the PLCγ inhibitor U-73122 does not affect bead internalization, as U-71322 has previously been shown to inhibit myelin endocytosis by CR3^-/- ^microglia [35] and PKC activation in response to oxidized LDL (oxLDL) [33]. However, the experimental conditions in these reports differ greatly from those described here as the authors use either primary murine microglia or LPS primed P388D_1 _cells. The signaling pathways and receptors utilized by these murine cells could be quite different from those utilized by our primary unprimed human GM-MØ. Furthermore, while PLCγ is an important activator of conventional PKC, atypical PLCγ-independent PKC isoenzymes have been shown to be important in a number of immune cell functions [70]. Our finding that PKC blockers inhibit internalization, but a PLCγ blocker does not, raises the possibility that GM-MØ utilize atypical PKC isoenzymes as second messenger signals for SR-mediated phagocytosis. While this has yet to be formally demonstrated, it is supported by our finding that an inhibitor of the atypical PKC isoenzyme activator PI-3K [70] blocks internalization.

Finally, the MAPK family of proteins are known to play an important role in MØ phagocytosis and have been implicated as downstream signaling molecules for SRs. Stimulation of SRs with fucoidan, oxLDL or poly(I) results in the activation of JNK and ERK MAPK pathways [37,31,38]. Furthermore, Lamprou and colleagues reported that inhibition of these pathways results in a reduction of latex bead internalization by medfly hemocytes [71,72]. The results of our experiments are consistent with these reports in that the inhibition of JNK and ERK pathways results in a reduction of bead internalization. This suggests that some of the pathways utilized during SR-mediated phagocytosis are conserved across a broad spectrum of species.

It is important to note that none of the signaling inhibitors tested in this report had any measurable effect on cell viability, size, density or bead binding. It is known that SR-A-mediated acetylated low density lipoprotein binding and cell adhesion require G proteins [73,74]. This, combined with the previous observation that particle binding by SRs is highly temperature dependent, suggests that it contains an active component. However, our data suggests that this active binding mechanism does not require actin filaments, microtubules, PKC, PI-3K, tyrosine kinases, MAPKs or PLCγ even though many of these pathways are necessary for internalization. Our finding that cytochalasin D has no effect on bead binding stands in contrast to the report of Post, *et al. *in which cytochalasin D was shown to inhibit SR-A-mediated cell attachment by 35% [73]. This discrepancy may reflect the differences between the cytoskeletal requirements for particle binding vs. firm anchorage to a substrate.

## Conclusion

We have developed a novel high-throughput assay for particle phagocytosis that we used to test the signaling pathways and cytoskeletal components required for unopsonized phagocytosis by human monocyte-derived MØ. We found that filamentous actin, microtubules, PKC, tyrosine kinases, PI-3K, MEK and JNK are required for optimal particle internalization while an inhibitor of PLCγ has no effect.

## Competing interests

The authors declare that they have no competing interests.

## Authors' contributions

THS conceived and designed the experiments, generated the biotin-conjugated beads and authored the manuscript. AI participated in experimental design and manuscript authoring and carried out the binding and phagocytosis assays. GD developed the custom data analysis software and participated in manuscript authoring. ARW generated the GM-MØ protocol and participated in manuscript revision. LK contributed to the conception and design of the experiments and played a significant role in the revision of the manuscript. All authors read and approved the final manuscript.
